# Chromosome-level genome assembly of *Cyperus iria*, an aggressive weed of rice

**DOI:** 10.1038/s41597-025-04470-6

**Published:** 2025-01-21

**Authors:** Siyu Yang, Qingyi Cao, Zexin Wen, Aoxue Wang, Shiyao Shi, Zhuoying Liang, Shuying Li, Wenjun Gui, Jinwen Zhu

**Affiliations:** 1 Institute of Pesticide and Environmental Toxicology, Zhejiang University, Hangzhou, 310058 P. R. China; 2 Ministry of Agriculture Key Laboratory of Molecular Biology of Crop Pathogens and Insect Pests, Zhejiang University, Hangzhou, 310058 P. R. China; 3 Key Laboratory of Biology of Crop Pathogens and Insects of Zhejiang Province, Zhejiang University, Hangzhou, 310058 China

**Keywords:** Genome, Plant genetics

## Abstract

*Cyperus iria* is an aggressive weed of rice throughout the world. Until now, the reference genome of *C. iria* has not been published. Here, we completed the chromosome-level genome assembly of *C. iria* based on Illumina, PacBio and Hi-C reads. The assembled genome size of *C. iria* was 479.08 Mb with a contig N50 of 7.02 Mb. 68 pseudochromosomes were produced using Hi-C scaffolding, accounting for 99.65% of the assembled genome. The number of predicted protein-coding genes is 47,395, of which 93.26% were annotated, and 37.69% repetitive sequences were identified. Our study provided a valuable genomic resource for the molecular biology research and the management of *C. iria*.

## Background & Summary

Agricultural weeds are a key constraint factor in agricultural production because they can compete with crops for light, nutrients, moisture and space, leading to decreased crop quality and yield^1^. Among all types of crop pests, weeds are known to cause the greatest reduction in crop yield^2^. As reported, the potential yield reduction caused by weeds could be up to 23%, 37%, 37%, 40%, 36% and 30% in wheat, soybeans, rice, maize, cotton and potatoes, respectively, with the average yield reduction of 34%, which is far more than the impacts of animal pests and pathogens^3^. With the increasing world population and decreasing available resources, weed management is a particularly important and challenging task.

From the evolutionary point, weeds is an excellent example of rapid adaptation to changing environments, due to their abundant genetic variation and plasticity, weeds can evolve more rapidly than crops^4,5^. Weeds have shown a remarkable capacity to quickly adapt to changing environmental factors, agricultural techniques, and weed-control strategies^6^. Understanding the evolution and adaptation of weeds is essential for effective weeds management. However, the genetic characterization of weed systems has received comparatively little attention^7^.

Genomic studies is an important approach to identify the origin of weed species and study their adaptive evolution, providing a basis for establishing effective weed management strategies^8^. However, despite the substantial impact of weeds on agricultural production, research on weeds have not received the necessary attention in both traditional molecular biology and genomic analyses^7,9^. To date, 2,847 plant species have been identified as weeds^10^. However, only approximately 26 species have been subjected to sequencing and *de novo* genome assembly^9^.

*Cyperus iria* (rice flatsedge), belonging to Cyperaceae family, is an annual sedge with a fibrous root system and C4 photosynthetic system^11^. It can reach 60 cm in height^12^. *C. iria* is native to tropical and subtropical countries^13^ with the ability to multiply rapidly and readily adapts to ecological niches^14^. The high seed production of *C. iria* (3000–5000 seeds per plant) combined with its short life cycle leads to a very high rate of reproduction^15,16^. *C. iria* is an aggressive weed throughout the world and has become a major agricultural weed in rice production systems in 22 countries^16,17^. It has been reported that a 64% reduction in rice yield is the result of *C. iria* infestation throughout the crop growth period^18^. Competition between *C. iria* and rice during the first 30 days can reduce the yield of rice by up to 12.9%, and infestation during the first 40 days can the yield of transplanted rice yield by up to 43.5%^19^. Chemical herbicides have become the preferred choice for *C. iria* control because it is highly efficient, less labour intensive, and cost effective. However, as a result of extensive herbicide use, *C. iria* has developed resistant populations to ALS (acetolactate synthetase) inhibitors, such as pyrazosulfuron-ethyl, halosulfuron-methyl and penoxsulam^20^. *C. iria* herbicidal resistance remains largely unknown at the molecular level. Therefore, understanding the function of *C. iria* genes and the mechanisms by which it has evolved to become invasive is critical to the management of this weed. In addition, as a traditional Chinese medicine, *C. iria* has been found to show promising pharmacological effects^12,13,21,22^. However, despite its medical importance, genomic and genetic information on *C. iria* is still very limited, severely hampering molecular and genetic research into this devastating weed.

Here, we generated a chromosome-level genome of the *C. iria* based on Illumina, PacBio and Hi-C technology. The assembled genome size of *C. iria* was 479.08 Mb (92.08% of the estimated genome size) with a contig N50 of 7.02 Mb. 99.65% of the assembled genome were anchored to 68 pseudochromosomes. The number of predicted protein-coding genes is 47,395, of which 93.26% were annotated, and 37.69% repetitive sequences were identified. The high-quality chromosome-level *C. iria* reference genome assembly, provides a strong basis for development of new strategies for the successful management of this aggressive weed in the future.

## Methods

### Plant material, library construction and sequencing

Seeds of the *C. iria* was collected from Huzhou, Zhejiang Province (30° 87′ N, 120° 10′ E) in 2021 and stored in 4 °C until used. Plants of *C. iria* were grown in small pots in the Experimental Greenhouse of Zhejiang University’s Zijingang Campus (30° 30′ N, 120° 08′ E) in Hangzhou, China, with 16 hours of light and temperatures of 25 °C at day and 20 °C at night. Fresh young leaves from the same one *C. iria* plant were collected and immediately frozen in liquid nitrogen. The cetyltrimethylammonium bromide (CTAB) method was employed to extract high quality, high molecular weight genomic DNA from young leaves^23^. The genomic DNA was used for the construction of an Illumina paired-end (PE) library with ∼450 bp insert sizes, and the libraries were constructed on an Illumina NovaSeq sequencing platform using Next-Generation Sequencing (NGS) following the standard procedure. For PacBio sequencing, the genomic DNA of *C. iria* was used for the construction of PacBio SMRTbell libraries according to the standard SMRTbell library preparation protocol, and the PacBio Sequel II platform was then utilized to sequence the libraries. The Hi-C library constructed from fresh young leaves of same one *C. iria* plant was then sequenced on an Illumina NovaSeq sequencing platform.

Total RNAs were extracted from four tissues (root, stem, leaf, and flower) of the same *C. iria* plant using TRIzol reagent. RNA from these four tissues was mixed equally for the construction of a PacBio Iso-Seq library. The cDNA was generated from mixed RNA using the SMARTer PCR cDNA Synthesis Kit (Clontech). The purified cDNA was then used to construct a Iso-Seq SMRTbell library using the SMRTbell Express Template Prep kit 1.0, which was subsequently sequenced on the PacBio Sequel II platform.

### Genome survey

**Fig. 1:**
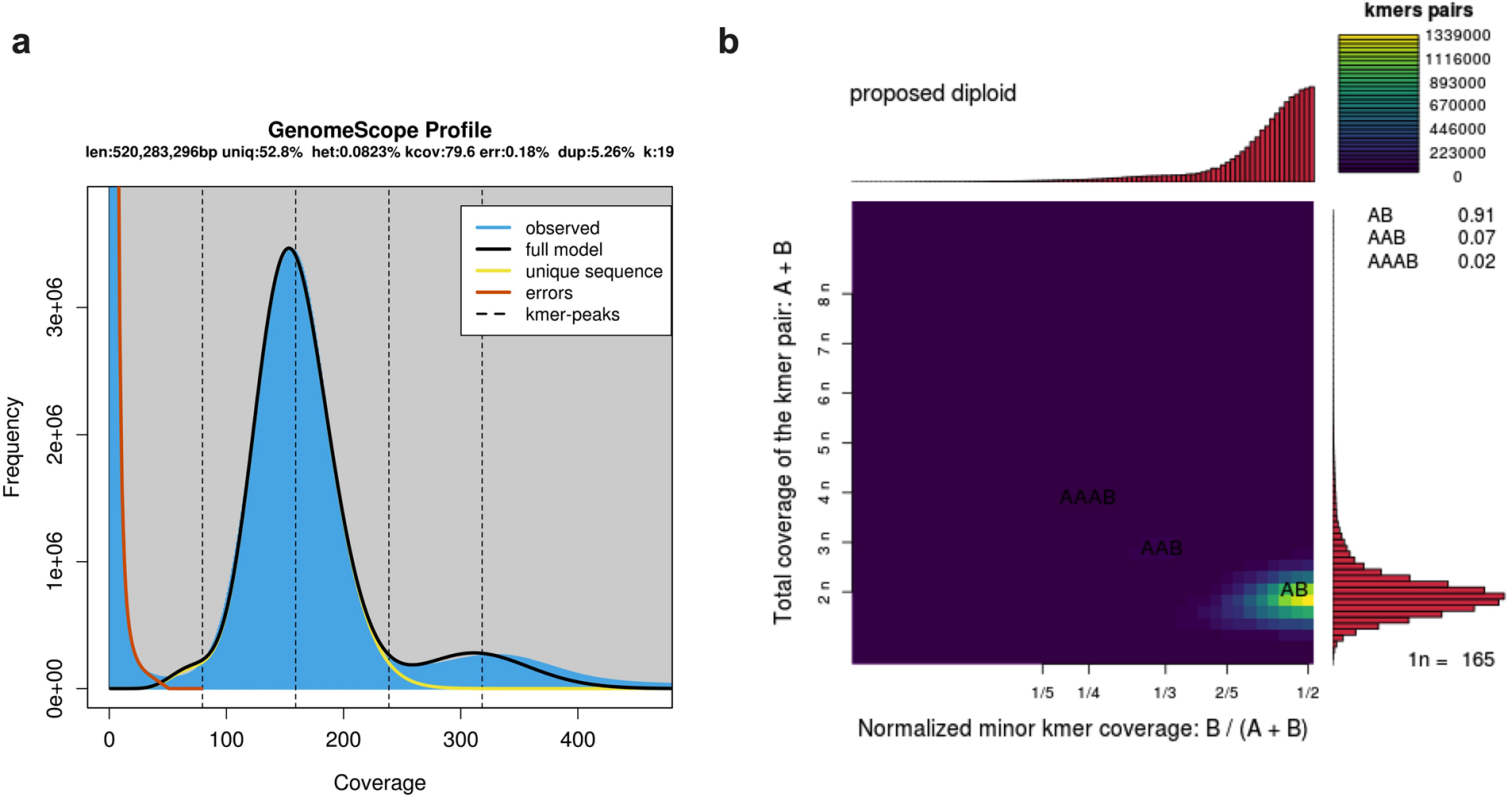
Genome size estimation and ploidy levels. (**a**) Estimation of genome size and heterozygosity based on k-mers (k = 19). (**b**) Ploidy inference based on Smudgeplot analysis.

After removing low-quality reads, 10,000 high-quality data pairs were randomly selected and mapped to the NCBI nucleotide (NT) database, revealing the top five matched species. Jellyfish software (version 2.3.0)^24^ was used for k-mer analysis of all high-quality data. Based on the k-mer frequency analysis (k-mer = 19), genome characteristics including genome size, heterozygosity and repeat rate, were estimated by using GenomeScope^25^. The genome size was calculated as following Eq. (1):

1 $$G=\frac{N\left(L-K+1\right)-B}{D}$$

wherein G represents the genome size, N means the total number of reads, L means the average length of reads, K is the k-mer length, and D is the peak depth that is estimated from the k-mer distribution (pkdepth). Low frequency k-mers before the first valley were discarded to minimise the influence of sequencing errors. The k-mer analysis (k-mer = 19) analysis determined the genome size to be 520.28 Mb, with the heterozygosity rate and repetitive fraction were 0.08% and 47.23%, respectively (Table S1 and Fig. 1a). These findings suggest that the genome of *C. iria* belongs to the simple genome.

Smudgeplot^26^ was used to estimate ploidy levels from corrected reads with a k-mer value of 19. The Smudgeplot performs gymnastics with heterozygous k-mer pairs extracted from k-mer count databases. It can disentangle genome structure by comparing the sum of coverages of k-mer pair (CovA + CovB) to their relative coverage (CovB/(CovA + CovB))^26^. Smudgeplot analysis indicates this genome assembly was a diploid (AB) with with a confidence of 0.91 (Fig. 1b).

### Genome assembly

To obtain a contig-level genome, PacBio High Fidelity (HiFi) reads were assembled using Hifiasm^27^. Totally 18.51 Gb Pacbio HiFi reads were generated, resulting in a genome assembly of 479.08 Mb with a contig N50 of 7.02 Mb and a GC content of 35.64% (Tables 1, 2). Purge_dups (version 1.2.5)^28^ was used to remove redundant sequences in the polished assembly. After that, only 80 contigs (462.83 Mb) remained for further analysis (Table 3). BWA (version 0.7.12)^29^ was used to compare the corrected high-quality Illumina reads sequence with the assembled genome sequence. The results showed that the read sequence comparison rate reached 99.25%, and the average sequencing depth was 215×. The sequence depth of 20× or more accounted for 99.78% (Table 4).

**Table 1:** Statistics of PacBio sequencing data of *C. iria* genome.

Items	Value
Total sequence number	1,003,630
Total sequence length (bp)	18,511,980,401
Min reads length (bp)	324
Max reads length (bp)	54,852
N50 (bp)	18,467

**Table 2:** Statistics of pre-assembly of *C. iria* genome.

Number of contig	Total length of contig (bp)	N50 of contig (bp)	Max contig (bp)	GC content (%)
369	479,081,043	7,015,313	10,521,918	35.64

**Table 3:** Statistics of pre-assembly of *C. iria* genome after redundancy removal.

Number of contig	Total length of contig (bp)	N50 of contig (bp)	Max contig (bp)	GC content (%)
80	462,829,887	7,104,101	10,521,918	35.53

**Table 4:** Statistics on the coverage of reads of *C. iria* genome.

Type	Items	Percentage (%)
Illumina Reads	Mapping rate	99.25
Genome	Average sequencing depth	215x
	Coverage ≥ 1	99.98
	Coverage ≥ 4	99.95
	Coverage ≥ 10	99.88
	Coverage ≥ 20	99.78

**Fig. 2:**
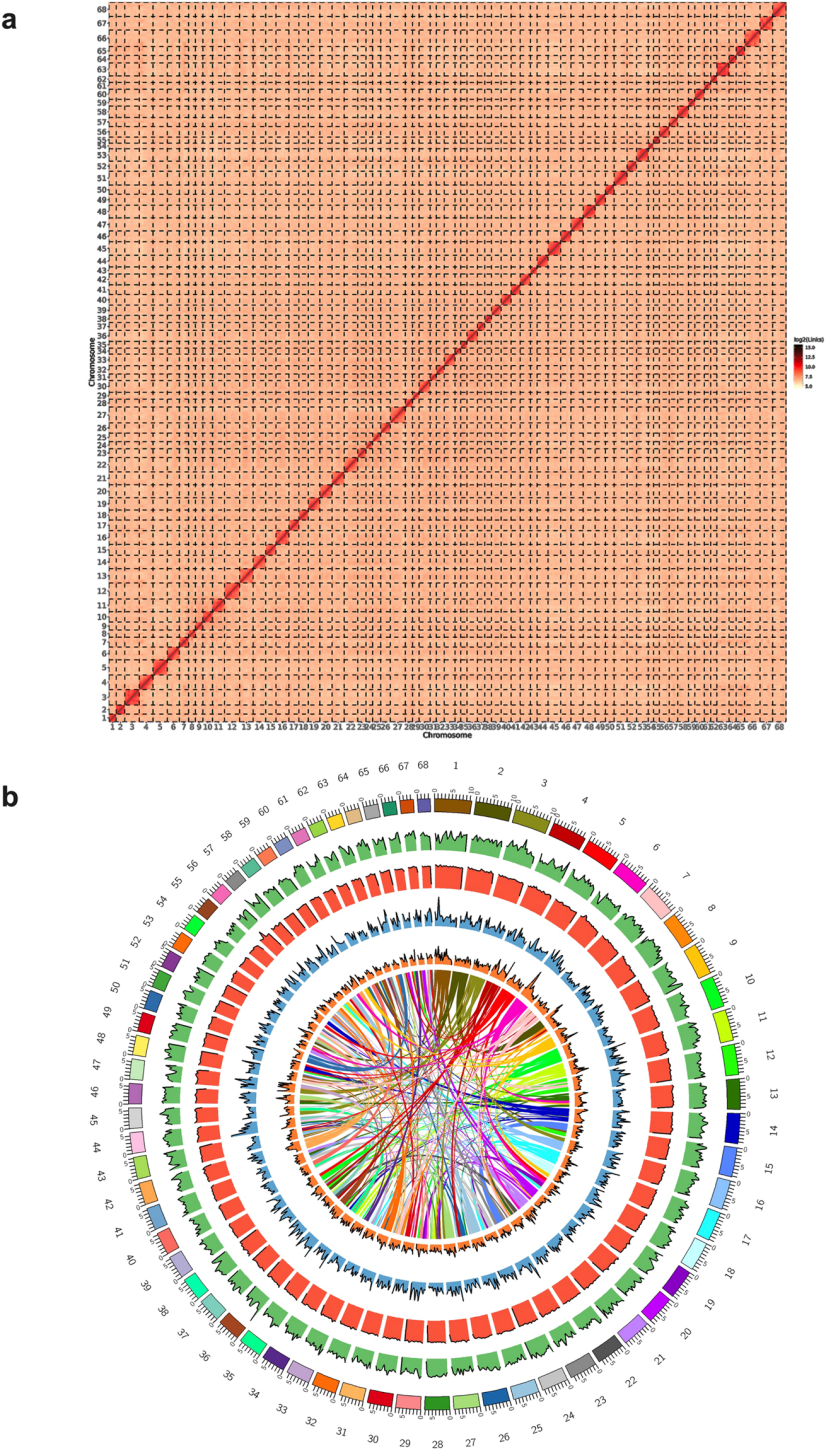
Overview of the *C. iria* genome assembly. (**a**) Hi–C interaction heatmap for *C. iria*. (**b**) Chromosomal features of *C. iria*. The circle diagram from the outside to the inside represents: Pseudochromosome, Gene density, GC content, LTR/Gopia transposon density, LTR/Gypsy transposable factor density. The connection lines in the circle are synteny lines of paralogous sequences in the genome.

To obtain a chromosome-level genome assembly of the *C. iria*, HiC-Pro software (version 3.1.0)^30^ was used to align clean Hi-C reads to the draft genome sequences in comparison mode. All invalid read pairs were removed using HiC-Pro software except unique mapped paired-ends, which were retained for further analysis. In total, Hi-C sequencing produced 65.73 Gb clean reads (Table 5), and 99.15% of Hi-C reads mapped to assembled contigs, including 65.30% unique mapped read pairs (Table 5). PacBio sequencing assemblies were organized into chromosome-level scaffolds by integrating valid interaction pairs from the unique mapped read pairs (Table 5). The ensuing Hi-C reads were then used to correct misjoins, order, orient, and anchor in the draft genome assembly with the 3D *de novo* assembly (3D-DNA) (version 201008)^31^. We used the Hi-C data to attach the draft genome to the chromosome level, 68 pseudochromosomes were constructed, whereby 99.65% of the assembled sequences were anchored. The heatmap of Hi-C interaction revealed a higher intensity of interactions in diagonals is higher than that in nondiagonal positions in each group, indicating the chromosome-level genome assembly was complete and robust (Fig. 2a). Chromosome lengths varied from 3,585,826 bp (Chr54) to 10,521,918 bp (Chr66) (Table S2 and Fig. 2b). Subsequently, the assembled results was polished using Racon (version 1.4.20)^32^. Benchmark for Universal Single Copy Orthologues (BUSCO, version 5.4.3)^33^ with the plant dataset (embryophyte) was used to assess the genome completeness and continuity. BUSCO analysis showed that 95.11% (0.50% fragmented and 4.40% missing BUSCOs) of the BUSCO genes in the *C. iria* genome were successfully identified as complete BUSCO, which indicates the high completeness of the genome assembly (Table S3). Furthermore, the contiguity of the genome was evaluated by calculating LTR Assembly Index (LAI) using LTR_retriever (version 2.9.9)^34^ with default parameters. The LAI value of the genome assembly was 9.77.

**Table 5:** Statistics of Hi-C data.

Statistics of Hi-C data				
Reads number	Total bases (bp)	Clean data (bp)	GC content (%)	Q30 (%)
444,906,852	66,736,027,800	65,729,426,903	38.35	93.66
**Statistics of mapping**				
Mapping type	Number of reads		Ratio (%)	
Total Paired-end Reads	221,141,266		100	
Mapped reads	219,258,960		99.15	
Unique mapped read pairs	144,412,361		65.30	
**Statistics of valid Hi-C data**				
Type		Number of reads		
Valid interaction pairs		116,080,912		
Dangling end pairs		17,732,887		
Re-ligation pairs		318,674		
Self-cycle pairs		862,843		
Dumped pairs		2,760

### Genome annotation

To identify repeat sequences, a combination of the homology-based prediction and *de novo*-based prediction was performed. For *de novo*-based prediction, RECON (version 1.0.8)^35^, RepeatModeler (version 2.0.4)^36^, and RepeatScout (version 1.0.6)^37^ were used to identified repetitive elements. For homology-based prediction, RepeatMasker (version 4.1.4)^38^ was used to identify repetitive elements by comparing to consensus sequences in the Repbase library^39^. The analysis of homology-based comparisons and *de novo*-based methods revealed that 37.69% of the *C. iria* genome consists of repetitive sequences, with LTRs representing 8.95% of the total. Gypsy was identified as the predominant LTR type (5.85%), followed by Copia (2.69%) (Table 6).

**Table 6:** Transposable elements in *C. iria* genome.

Repeat type	Number of elements	Length (bp)	Percentage in Genome (%)
**Retrotransposons**	**67,375**	**46,178,271**	**9.98**
LTR-Retrotransposons	53,817	41,428,342	8.95
LTR/Copia	16,111	12,438,150	2.69
LTR/Gypsy	33,640	27,056,182	5.85
LTR-other	1,263	870,280	0.19
Non-LTR Retrotransposons	13,558	4,749,929	1.03
LINE	13,527	4,746,199	1.03
SINE	31	3,730	0.00
**DNA Transposons**	**79,325**	**22,334,102**	**4.83**
DNA/hobo-Activator	17,494	8,213,528	1.77
DNA/Tcl-IS630-Pogo	1,436	250,320	0.05
DNA/En-Spm	0	0	0.00
DNA/MULE-MuDR	16,106	5,311,897	1.15
DNA/PiggyBac	0	0	0.00
DNA/Harbinger	2,422	698,970	0.15
**Rolling-circles**	**12,328**	**1,444,953**	**0**.**31**
**Unclassified**	**529,888**	**105,928,532**	**22**.**89**
**Total interspersed repeats**	**133,010**	**37,459,884**	**10**.**51**
**Satellites**	**591**	**206,892**	**0**.**04**
**Simple repeats**	**7,496**	**440,263**	**0**.**10**
**Low complexity**	**0**	**0**	**0**

Non-coding RNA (ncRNA) species, such as rRNA, tRNA, miRNA, and snRNA, were annotated with RNAmmer (version 1.2) (for rRNAs prediction)^40^, tRNAscan-SE (version 1.3.1) (for tRNAs prediction)^41^, and Perl program Rfam (version 1.0) (for other two non-coding RNAs prediction)^42^. Totally 2,355 ncRNA genes were also identified, which included 599 rRNA, 985 tRNA and 771 other ncRNA (Table 7).

**Table 7:** The statistical results of non-coding RNA of *C. iria*.

ncRNA Type	Copy	Avg. length (bp)	Total length (bp)	% of genome
8 s rRNA	536	114.98	61,631	0.0133
18 s rRNA	32	1,762.59	56,403	0.0122
28 s rRNA	31	4,127.00	127,937	0.0276
tRNA	985	73.62	72,518	0.0157
other ncRNA	771	131.08	101,064	0.0218

To predict protein-coding genes, a combination of homology-based, *de novo*-based, and transcript-based prediction approaches was used. The *de novo*-based prediction was conducted with Augustus (version 3.3.2)^43^, GlimmerHMM (version 3.0.4)^44^, GeneMark (version 4.35)^45^, and GeneID (version 1.4) with default parameters. The homology-based prediction was performed using Exonerate (version 2.2.0)^46^ together with protein sequences from *Carex littledalei*, *Arabidopsis thaliana* and *Oryza sativa*. For transcript-based prediction, the high-quality full-length transcript data were compared and spliced using PASA (version 2.5.2)^47^ to obtain the corresponding gene prediction results. Finally, EvidenceModeler (version r2012-06-25)^48^ was used to integrate homology-based, *de novo*-based, and transcript-based prediction results. The prediction results revealed that *C. iria* genome contained 47,395 protein-coding genes, and the average gene length is 2,762.5 bp (Table 8). The BUSCO analysis evaluated the gene set completeness, revealing that 96.28% (1,554 genes) of the BUSCO genes were present in the *C. iria* gene set. This finding confirms the high quality of gene prediction. BLASTP (version 2.0.14.152) (E-value < 1 × 10^−5^) searches against the NR and SwissProt databases were performed for the final protein-coding, functionally annotated genes. Functional domains were then obtained by searching publicly available databases using InterProScan (version 5.61–93.0)^49^. GO annotation of protein-coding genes was performed using interproscan. The KEGG pathway annotation of protein-coding genes was mainly performed by KEGG Automatic Annotation Server (KAAS, version 2.1)^50^. Approximately 93.26% of the protein-coding genes were functionally annotated (Table 9). To further validate the accuracy of our gene annotations, we compared the gene sequences with transcript sequences using geneBody_coverage (version 5.0.1)^51^. This analysis assessed the sequence coverage across the entire gene length, from the 5′ to the 3′ end, to determine if the sequencing reads were evenly distributed. Figure S1 illustrates the alignment results, demonstrating that the sequencing reads were uniformly distributed without bias towards the 5′ or 3′ ends. The mapping rate of transcript sequences to the gene sequences was 93%, further supporting the accuracy of the gene annotations.

**Table 8:** The statistical results of gene prediction of *C. iria*.

Property	Value
Total Genes length (bp)	130,929,161
Genes Percentage of genome	28.289%
Total Genes Number	47,395
Average gene length (bp)	2,762.5
Total Exons Number	248,303
Average Exons Per Gene	5.2
Total Exons length (bp)	56,684,235
Exons Percentage Of genome	12.2474%
Total CDS Number	248,303
Average CDS length (bp)	228.2
Average Exons length (bp)	228.2
Average Introns length (bp)	369.5
Total CDSs length (bp)	56,684,235
CDSs Percentage of genome	12.2474%
Average transcription length (bp)	1,195.9

**Table 9:** The statistical results of gene function annotation of *C. iria*.

Annotation in Database	No. Of Genes	Percentage %
GO	28,561	60.26
KEGG	12,499	26.37
Swissport	33,014	69.66
NR	44,115	93.08
At least one database	44,199	93.26

## Data Records

Raw Illumina, PacBio HiFi and Hi-C of *C. iria* genome sequencing data were deposited in the NCBI BioProject database under project accession number PRJNA1157994^52^, with accession numbers SRR30588109^53^ for Illumina sequencing data, SRR30588108^54^ for Pacbio sequencing data, SRR30588107^55^ for Hi-C sequencing data, SRR30588106^56^ for Iso-Seq data. The genome assembly has been deposited at GenBank under the accession JBHOFI000000000^57^. The annotation of the *C. iria* genome has been submitted to the online open-access repository Figshare^58^ database.

## Technical Validation

BWA (version 0.7.12)^29^ was used to compare the corrected high-quality Illumina reads sequence with the assembled genome sequence. The read sequence comparison rate reached 99.25%, and the average sequencing depth was 215×. The sequence depth of 20× or more accounted for 99.78% (Table 4). Benchmark for Universal Single Copy Orthologues (BUSCO, version 5.4.3)^33^ with the plant dataset (embryophyte) was used to assess the genome completeness and continuity. BUSCO analysis showed that 95.11% (0.50% fragmented and 4.40% missing BUSCOs) of the BUSCO genes in the *C. iria* genome were successfully identified as complete BUSCO, which indicates the high completeness of the genome assembly (Table S3). Then, the assembly continuity was determined by analyzing the LTR Assembly Index (LAI), the LAI score was 9.77. The heatmap of Hi-C interaction revealed a higher intensity of interactions in diagonals is higher than that in nondiagonal positions in each group, indicating the chromosome-level genome assembly was complete and robust (Fig. 2a). The BUSCO analysis also used to evaluate the gene set completeness, revealing that 96.28% (1,554 genes) of the BUSCO genes were present in the *C. iria* gene set. This finding confirms the high quality of gene prediction.

## Supplementary information

Supplementary Information

## Data Availability

The pipeline and software utilized in this study were employed for data analysis following manual instructions and protocols. Details on the software version and parameters are outlined in the Methods section. In cases where specific parameters are not specified, default settings were applied.
