# Supplementary material for: Chromosome-level genome assembly of Cyperus iria, an aggressive weed of rice
Source: Sci Data. 2025 Jan 21;12:120. doi: 10.1038/s41597-025-04470-6 (PMC11751304; doi:10.1038/s41597-025-04470-6)
Supplement: Supplementary file 1 — Supplementary Information [file 41597_2025_4470_MOESM1_ESM.pdf]

Supplementary Information

Chromosome-level genome assembly of *Cyperus iria*, an aggressive weed of rice

Siyu Yang[1], Qingyi Cao[1], Zexin Wen[1], Aoxue Wang[1], Shiyao Shi[1], Zhuoying Liang[1], Shuying Li[1], Wenjun Gui[1,2,3**], Jinwen Zhu[1*]

1. Institute of Pesticide and Environmental Toxicology, Zhejiang University, Hangzhou 310058, P. R. China
2. Ministry of Agriculture Key Laboratory of Molecular Biology of Crop Pathogens and Insect Pests, Zhejiang University, Hangzhou, 310058, PR China
3. Key Laboratory of Biology of Crop Pathogens and Insects of Zhejiang Province, Zhejiang University, Hangzhou, 310058, China

corresponding authors: Jinwen Zhu (zhjw@zju.edu.cn); Wenjun Gui (guiwj@zju.edu.cn)

Contents

Title	Page
Table S1	2
Table S2	2
Table S3	3
Fig. S1	4

Table S1. Statistics of 19-mer analysis of *C. iria*.

K-mer depth	Genome size (Mb)	Heterozygosity (%)	Repetitive fraction (%)
154	520.28	0.08	47.23

Table S2. The length of each chromosome of *C. iria* genome

Chr	Length
chr1	4,903,511
chr2	5,828,797
chr3	9,886,634
chr4	9,360,301
chr5	9,718,029
chr6	8,208,398
chr7	6,322,831
chr8	4,599,740
chr9	5,105,191
chr10	6,607,252
chr11	8,153,141
chr12	10,272,165
chr13	9,194,722
chr14	8,577,497
chr15	6,988,139
chr16	8,820,644
chr17	6,780,524
chr18	6,404,754
chr19	7,879,065
chr20	8,324,110
chr21	8,239,960
chr22	9,233,046
chr23	5,616,093
chr24	4,516,109
chr25	5,504,278
chr26	6,535,687
chr27	10,204,745
chr28	5,005,569
chr29	4,392,874
chr30	7,540,048
chr31	4,202,028
chr32	5,391,343
chr33	7,367,240
chr34	3,858,859
chr35	4,285,298
chr36	7,104,101
chr37	4,993,775

Chr	Length
chr38	4,582,656
chr39	6,590,063
chr40	6,761,857
chr41	6,212,755
chr42	6,955,261
chr43	4,552,899
chr44	7,457,113
chr45	8,649,576
chr46	6,966,917
chr47	8,317,637
chr48	8,150,531
chr49	7,015,313
chr50	5,908,151
chr51	8,831,915
chr52	6,456,454
chr53	7,979,095
chr54	3,585,826
chr55	3,910,112
chr56	6,763,563
chr57	5,781,640
chr58	7,333,483
chr59	4,328,824
chr60	6,440,147
chr61	4,380,798
chr62	4,159,076
chr63	8,322,859
chr64	4,821,552
chr65	5,761,770
chr66	10,521,918
chr67	8,644,607
chr68	9,132,232

Table S3. Genome completeness and continuity assessment.

Groups	Numbers	%Completeness
Complete BUSCOs	1,535	95.11%
Complete and single-copy BUSCOs	305	18.90%
Complete and duplicated BUSCOs	1,230	76.21%
Fragmented BUSCOs	8	0.50%
Missing BUSCOs	71	4.40%
Total BUSCO groups searched	1,614	100%

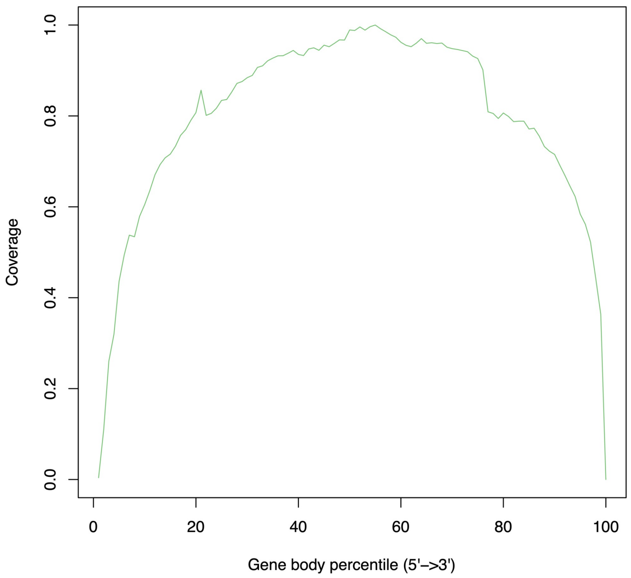

Fig. S1. Gene Coverage Uniformity Analysis: Alignment of Transcriptome Data to Genome. The x-axis represents the percentage of gene length from 0% (5' end) to 100% (3' end), and the y-axis represents the total number of aligned sequences reads normalized by region.
